# Cocaine-Induced Bilateral Internal Carotid Artery Dissection: A Case Report

**DOI:** 10.7759/cureus.48548

**Published:** 2023-11-09

**Authors:** Kawthar F Abdulnabi, Warda F Alalawi, Sayed Mohamed S Ahmed, Kawthar M Abdulla, Ahmad A Abdultawab

**Affiliations:** 1 General Practice, First Moscow State Medical University, Moscow, RUS; 2 General Practice, Misr University for Science and Technology, 6th of October City, EGY; 3 Emergency Medicine, Dallah Hospital, Riyadh, SAU

**Keywords:** case report, internal carotid artery, arterial dissection, substance abuse, cocaine use

## Abstract

Internal carotid artery dissection is a rare vascular condition with potentially life-threatening consequences, often resulting in intramural hematomas and luminal narrowing. Various etiological factors, including cocaine use, contribute to its occurrence. This case report explores a striking instance of bilateral internal carotid artery dissection in a middle-aged man with a history of chronic cocaine use, shedding light on the intricate relationship between substance abuse and vascular pathology. We present the case of a 47-year-old man with a significant history of chronic cocaine use presented with sudden-onset severe headaches and visual disturbances, including blurred vision and diplopia. Physical examination revealed signs of Horner's syndrome and neurological involvement. Diagnostic imaging confirmed bilateral internal carotid artery dissections, primarily on the right side, with mural hematoma formation and luminal narrowing. Immediate management included pain control, blood pressure regulation, and discontinuation of cocaine use. The patient's symptoms gradually resolved with anticoagulation therapy, and he was discharged with a comprehensive follow-up plan. This case of bilateral internal carotid artery dissection in a middle-aged man with a history of chronic cocaine use underscores the intricate relationship between this condition and substance abuse. It highlights the need for a comprehensive clinical history to identify potential links between substance use and vascular pathologies. The multidisciplinary approach to diagnosis and management is crucial in optimizing patient outcomes. Addressing substance abuse as a contributing factor to vascular pathology is essential, emphasizing the importance of comprehensive care and support for affected individuals, and contributing valuable insights to the existing literature on vascular pathology.

## Introduction

Internal carotid artery dissection is a rare and potentially life-threatening vascular condition characterized by the separation of the arterial wall layers, often leading to the formation of intramural hematomas and luminal narrowing. It is an infrequent yet critical cause of stroke, especially in young to middle-aged individuals. Internal carotid artery dissection is associated with various etiological factors, including trauma, connective tissue disorders, and, notably, the use of cocaine [1]. This case report describes an instance of bilateral internal carotid artery dissection in a middle-aged man, with a significant history of chronic cocaine use, shedding light on the intricate interplay between substance abuse and vascular pathology.

Cocaine, a potent sympathomimetic drug, exerts multifaceted effects on the cardiovascular system, including arterial vasoconstriction, elevated systemic blood pressure, and increased shear stress on arterial walls. Such hemodynamic changes may contribute to the development of arterial dissections [2,3]. Despite the rarity of internal carotid artery dissection, early diagnosis and management are paramount to prevent devastating complications, including ischemic stroke. This case emphasizes the clinical challenges in diagnosing and treating internal carotid artery dissection in the context of cocaine use and underscores the need for comprehensive evaluation and a multidisciplinary approach to patient care.

## Case presentation

We present the case of a 47-year-old man with a significant history of cocaine use. He presented to the emergency department with a sudden onset of severe headaches and visual disturbances, specifically experiencing blurred vision and diplopia. The patient reported a history of chronic cocaine use for several years, which he initially denied but later disclosed during the medical interview. There was no documented history of hypertension, hyperlipidemia, or previous vascular events.

Upon physical examination, the patient's vital signs were within normal limits. He exhibited signs of Horner's syndrome, with ptosis of the right eyelid, miosis, and facial anhidrosis, suggesting sympathetic dysfunction. There was also a marked reduction in visual acuity, particularly in the right eye, and subtle facial asymmetry. These findings indicated neurological involvement.

Routine blood tests, including complete blood count, electrolytes, and renal and liver function tests, were within normal limits. Cocaine metabolites were detected in the patient's urine, confirming recent drug use. Given the patient's clinical presentation, the differential diagnosis included causes of sudden-onset headache and visual disturbances, such as migraines, hypertensive crisis, and posterior circulation strokes.

**Figure 1:**
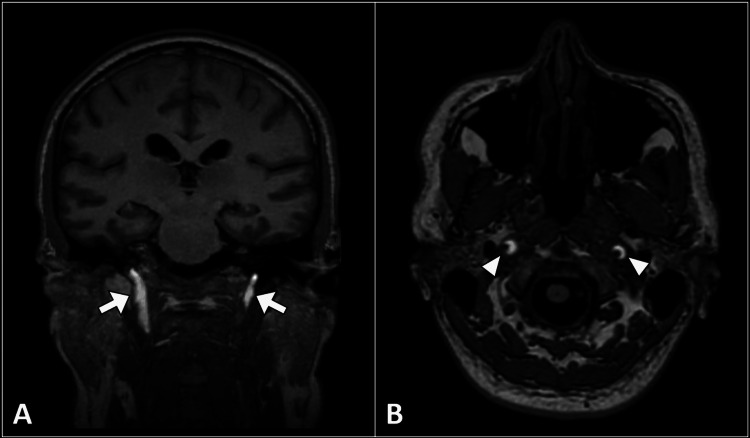
T1-weighted MR images in coronal (A) and axial (B) planes reveal bilateral acute hematomas in the internal carotid arteries (arrows). Notably, the crescent sign is depicted (arrowheads), indicative of vascular dissection. MR: magnetic resonance

**Figure 2:**
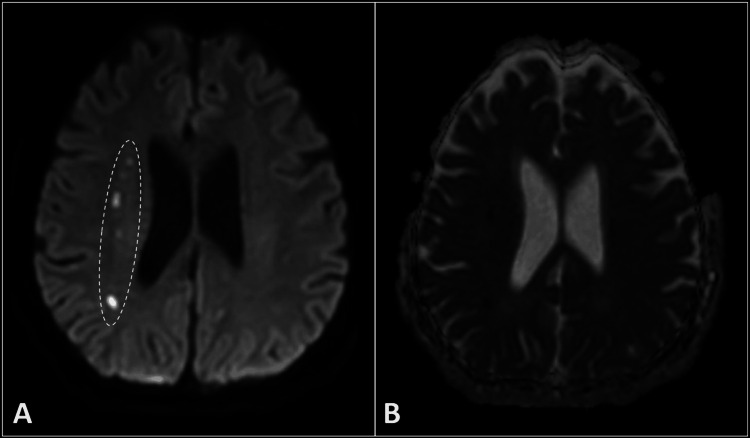
DWI (A) along with its corresponding ADC map (B) illustrates regions of restricted diffusion in the deep watershed areas (encircled) of the right cerebral hemisphere, consistent with acute infarction. DWI: diffusion-weighted image, ADC: apparent diffusion coefficient

The patient underwent magnetic resonance imaging of the brain, which revealed bilateral internal carotid artery dissections, predominantly involving the right side. The dissections were characterized by mural hematoma formation, significant luminal narrowing, and irregular vessel walls. Acute watershed infarcts were noted in the right cerebral hemisphere as evident on the diffusion-weighted imaging. No other intracranial pathology, such as aneurysms or thrombosis, was identified (Figures 1-2). The definitive diagnosis was bilateral internal carotid artery dissection, as confirmed by magnetic resonance angiography. The patient's history of cocaine use was a significant contributing factor to the development of this rare vascular condition.

Immediate management involved pain control with analgesics, and blood pressure control with antihypertensive medication, including both alpha and beta blockers. Given the high risk of thromboembolic events in carotid dissection, the patient was anticoagulated with heparin and subsequently transitioned to oral anticoagulation with warfarin. Close monitoring of neurological deficits and radiological assessments was initiated.

The patient's hospital course was characterized by the gradual resolution of his symptoms, including the disappearance of Horner's syndrome and improved visual acuity. The patient remained on anticoagulation therapy and was educated on the importance of cessation of cocaine use and control of vascular risk factors. The patient was discharged with a comprehensive plan for outpatient follow-up, including regular visits with a neurologist and vascular specialist to monitor his progress, optimize anticoagulation therapy, and address long-term management and risk factor modification.

## Discussion

Internal carotid artery dissection, a rare but critical vascular pathology, presents distinctive challenges, particularly when linked to chronic cocaine use. The case of a 47-year-old man described here exemplifies the relationship between this condition and substance abuse, with notable clinical implications.

The association between bilateral internal carotid artery dissection and cocaine use in this case underscores the complexities involved in identifying contributory factors in vascular events. Cocaine, a potent sympathomimetic drug, exerts a range of cardiovascular effects, including arterial vasoconstriction, increased systemic blood pressure, and elevated shear stress on arterial walls. These hemodynamic changes render individuals susceptible to arterial dissections [1,4]. This case highlights the need for a comprehensive clinical history to uncover potential links between substance use and vascular pathologies.

Diagnostic advances, especially in radiological imaging, have enhanced our ability to detect and assess internal carotid artery dissection. Computed tomography angiography played a pivotal role in diagnosing bilateral dissections. Its high specificity and sensitivity provide a detailed evaluation of arterial morphology [5]. Additionally, magnetic resonance imaging, incorporating magnetic resonance angiography and axial fat-suppressed T1-weighted images, facilitated a comprehensive assessment, particularly for the presence of intramural hematoma, a significant diagnostic indicator [5]. Notably, the "crescent sign" has critical diagnostic value, signifying the acute nature of the dissection [4,5].

Management of internal carotid artery dissection necessitates a multidisciplinary approach [1,5]. The initial intervention typically involves medical management with anticoagulation therapy and antiplatelets, given the self-healing nature of most dissections. In cases involving cocaine-induced hypertension, the combined use of alpha and beta-blockers, as observed in this patient's treatment, is imperative. Managing patients with cocaine-related chest pain presents unique challenges, and the literature highlights the importance of balanced alpha and beta-blockade to mitigate arterial vasoconstriction and minimize the risk of worsening myocardial ischemia [2,4].

While percutaneous intervention and stent placement are options, they are reserved for cases where medical management proves inadequate or when symptoms become refractory due to the risk of restenosis. The high rate of spontaneous healing observed in internal carotid artery dissection patients aligns with our case, where the patient showed gradual clinical improvement under medical management [4,5].

## Conclusions

In conclusion, the case of bilateral internal carotid artery dissection in a middle-aged man with a history of chronic cocaine use sheds light on the complexities involved in diagnosing and managing such cases. Adherence to a multidisciplinary approach and a high index of suspicion are pivotal in optimizing patient outcomes. This case emphasizes the importance of addressing substance abuse as a contributory factor to vascular pathology and underscores the need for comprehensive care and support for affected individuals. The valuable insights from this case contribute to the growing body of knowledge on internal carotid artery dissection and its associated risk factors, making it a valuable addition to the existing literature on vascular pathology.
